# LiCl@AC Composites for Atmospheric Water Harvesting: Effect of the Salt Content

**DOI:** 10.1021/acsomega.5c06858

**Published:** 2025-11-05

**Authors:** Sonia Judith Segovia-Sandoval, Antonio García-Ripoll, Judit Farrando-Perez, Coset Abreu-Jauregui, Joaquin Silvestre-Albero

**Affiliations:** Laboratorio de Materiales Avanzados, Departamento de Química Inorgánica-Instituto Universitario de Materiales, 16718 Universidad de Alicante, Alicante 03690, Spain

## Abstract

Freshwater scarcity is a major global challenge threatening human well-being. Sorption-based atmospheric water harvesting using composites of porous materials and hygroscopic salts, offers a promising solution. In this study, activated carbon (AC) with a well-developed porous structure was impregnated with different loadings of LiCl to enhance its water sorption capacity. The resulting LiCl@AC composites were characterized in terms of their morphological, thermal, physicochemical, and textural properties, and their water sorption performance was evaluated under various relative humidity (RH) conditions and cycling tests. The composite containing 30 wt % LiCl (30LiCl@AC) exhibited the highest water uptake (0.48 g/g at 60% RH and 25 °C), approximately 4 times greater than pristine AC. This enhancement is attributed to the synergistic interaction between the hydrophilic LiCl and the hydrophobic AC surface. Adsorption–desorption cyclability tests revealed that the minimum regeneration temperature is 80 °C.

## Graphical abstract

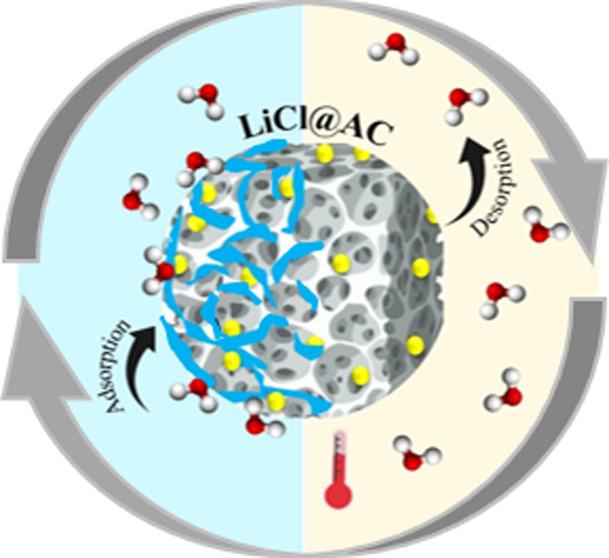

## 1 Introduction

Freshwater scarcity has become one of the most urgent challenges facing the Mediterranean region, driven by recurrent droughts and rising demand for water resources.^1–3^ Over the past decade, the region has experienced severe rainfall reductions, leading to low river flows, reservoir levels at only 20–30% of capacity, and widespread water shortages.^4^ The agricultural sector has been particularly affected: in Spain, for example, production fell by 13.6% in 2022—three times higher than the EU average—mainly due to drought-related stress on crops and soils.^5^ Some regions, such as Catalonia and Andalusia, already experience winter water restrictions, a worrying sign as extreme summer heatwaves become more frequent. Population growth along the Mediterranean coast and the intensification of heat events are expected to aggravate the crisis in the coming decades.

Traditional approaches—such as seawater desalination, wastewater reuse, and interbasin transfers—have been implemented to mitigate shortages.^6,7^ However, these technologies remain energy-intensive, capital-demanding, and often associated with environmental concerns. A promising complementary strategy lies in atmospheric water harvesting (AWH), which captures moisture directly from air.^8^ Given average Mediterranean conditions of 25 °C and 60% relative humidity (RH), the water content in the air could reach up to 8.8 g/kg (≈11.3 mL/m^3^). For a city like Alicante (200 km^2^), considering an air height of 1 km, this represents roughly 2.3 million m^3^ of potential water—about 1/20th of the daily discharge of the Ebro River. Globally, the atmosphere holds an estimated 12,900 trillion liters of water in vapor, cloud, and fog form.^9^

Among AWH technologies, sorption-based systems have attracted growing interest for their low energy demand, and potential for decentralized water supply. Their performance depends largely on the properties of the sorbent materials, which must efficiently capture and release water even under medium to low humidity (<60% RH).^10^ To compete with conventional technologies, these systems require minimal maintenance, low energy requirement, small environmental footprint, high capture capacity, and strong cycling stability. Materials investigated for this purpose include silica gel, zeolites, hygroscopic salts, activated carbons, hydrogels, and metal–organic frameworks (MOFs).^11,12^

Silica gel is an affordable and available material but exhibits limited water uptake (0.05 g/g at 30 °C) and poor desorption capability and limited thermal stability.^13^ MOFs, while tunable and highly porous,^14^ face challenges related to complex synthesis, high cost, and toxicity concerns.^15–18^ For instance, MOF-801 achieved 0.40 g/g at 90% RH; 0.28 g/g at 20% RH,^19^ while MOF-303, MOF-313, MIL-160, CAU-23, CAU-10, and Al-fumarate exhibit values of approximately 0.38–0.40 g/g at 40% RH.^20^

Hygroscopic salts (LiCl, CaCl_2_, LiBr) offer high capacities and low cost, but their use is hindered by aggregation and deliquescence at high RH, which leads to loss of salt and reduce stability.^21–23^ Zeolites (e.g., AQSOA type) display high uptake (0.26 g/g at 20% RH) but suffer from limited regenerability due to strong water binding.^24^ Hydrogels, characterized by 3D cross-linked polymer networks, can absorb significant amounts of water, but conventional hydrogels typically exhibit low surface area, and slow adsorption rates.^25,26^

Nature provides inspiration for improving these systems. Desert beetles and cactus spines combine hydrophilic regions that capture water with hydrophobic surfaces that guide droplets for collection. Mimicking this synergy, dispersing hygroscopic salts (LiCl, CaCl_2_, LiBr) within high-surface-area hydrophobic matrices—such as AC—can integrate both functionalities. Fine salt dispersion enhances surface-to-volume ratio, improves stability by limiting aggregation, reduces deliquescence losses, and facilitates water desorption.

Successful incorporation of hygroscopic salts has been reported in silica gels,^27^ porous carbons,^28^ zeolites,^29^ MOFs,^8^ and hydrogels.^30^ For instance, Xu et al. increased the water uptake of MIL-101­(Cr) from <0.1 g/g (pure) to 0.33 g/g and 0.77 g/g with 33 and 51 wt % LiCl, respectively.^8^ Li et al. synthesized a hybrid PAM/CNT/CaCl_2_ hybrid sorbent achieving 1.1 g/g at 60% RH.^30^

Despite these advances, key parameters—such as optimal salt content, regeneration temperature, and long-term cycling behavior—remain insufficiently explored. The aim of this work is to investigate high-surface-area activated carbon (AC) as a platform for the dispersion of hygroscopic LiCl and evaluates its performance for atmospheric water harvesting. The study examines the effects of LiCl loading, regeneration temperature, and cycling stability under four humidity conditions (30%, 40%, 60%, and 90% RH). The performance of LiCl-based composites is further compared with two benchmark adsorbents: zeolite 13× and the MOF UiO-66.

## 2 Materials and Methods

### 2.1 Materials

RGC-30 granular activated carbon, sourced from Nuchar, was selected as the platform due to its high surface area (above 1300 m^2^/g), and well-developed micro- and mesoporous structure. LiCl salt was used as the hygroscopic salt (anhydrous, 99%, Sigma-Aldrich). Zeolite 13× (spherical particles, 1.6–2.6 mm) was supplied by ZEOCHEM, and UiO-66 was synthesized in the laboratory following the procedure described by Katz et al.,^31^ with further details provided below.

### 2.2 Preparation of LiCl@AC Composites

RGC-30 granular activated carbon (AC) was dried at 150 °C for 12 h. Then, the required amount of LiCl was dissolved in 7 mL of distilled water to achieve the desired concentrations (5%, 10%, 15%, and 30 wt %) and was placed in contact with 2 g of AC for 12 h at room temperature (RT) under continuous stirring. After 12 h of impregnation, the samples were filtered under vacuum using a Büchner funnel with Whatman No. 1 filter paper (pore size ∼11 μm). The resulting solid was then collected and dried overnight at 150 °C, Figure S1a. The composites were designated as *X*LiCl@AC (*X* = 5%, 10%, 15%, and 30%), where *X* represents the nominal LiCl loadings.

Briefly, UiO-66 sample was prepared by dissolving 0.5 g of ZrCl_4_ in 20 mL of dimethylformamide (DMF) and 4 mL of concentrated HCl. Simultaneously, 0.492 g of terephthalic acid (DBC) was dissolved in 40 mL of DMF in a separate vessel. The two solutions were then combined and continuously stirred for 30 min. The resulting colorless solution was transferred to a tightly sealed 200 mL screw-capped glass vial and heated at 80 °C overnight. The solid product was filtered and rinsed two times with DMF (30 mL) and two times with ethanol (30 mL). Finally, the sample was activated by outgassing at 150 °C for 3 h under ultrahigh vacuum conditions.^31^

### 2.3 Characterization of LiCl@AC Composites

Textural parameters of the prepared composites were evaluated by N_2_ adsorption/desorption isotherms at cryogenic temperatures (−196 °C). The physisorption measurements were obtained with fully automated manometric equipment, designed and developed by the LMA group at the University of Alicante. Prior to the measurements, the samples were outgassed at 150 °C for 12 h under ultrahigh vacuum conditions. The specific surface areas (*S*_BET_) were estimated using the BET equation, while the total pore volume (*V*_total_) was determined from the amount of N_2_ adsorbed at *P*/*P*_0_ = 0.97. The micropore volume (*V*_micro_) was obtained using the Dubinin–Radushkevich (DR) method, and the mesopore volume (*V*_meso_) was calculated as the difference between *V*_total_ – *V*_micro_. Pore size distribution (PSD) was determined by DFT method. The thermogravimetric analysis was performed using a Mettler Toledo TGA/SDTA equipment. A 10 mg sample was placed in an alumina crucible and heated to 1025 °C at a rate of 10 °C per minute, under a N_2_ atmosphere with a gas flow rate of 100 mL/min. The crystal phases of the composites were analyzed by X-ray diffraction (XRD) using a Bruker D8-ADVANCE instrument. XRD patterns were recorded between 10° and 60° (2θ) with a step of 0.05°. Fourier Transform Infrared Spectroscopy (FTIR) measurements were performed in a JASCO FTIR 4700 spectrometer with a resolution of 2 cm^–1^ a Germanium encapsulated KBr beam splitter, and DLaTGS detector. The surface morphology of the samples was evaluated using field-emission scanning electron microscopy (FESEM). These analyses were performed in an IT500HR/LA system from JEOL with a resolution of 1.5 nm at 30 kV and 4 nm at 1 kV. Energy-disperse X-ray spectroscopy (EDS) was used to analyze the elemental distribution of C, O, and Cl. To quantify Li content in the composites, the composite samples were digested using the Ultraware (UW) microwave digestion system with an acid mixture (HNO_3_ and HCl). The digests were diluted with ultrapure water and analyzed then by Inductively Coupled Plasma-Optical Emission Spectrometry (ICP-OES) using a PerkinElmer equipment, model Optima7300 DV. The obtained results are expressed as mg of elemental Li per kg of composite. Immersion calorimetry measurements were performed at 30 °C using a Tian-Calvet microcalorimeter (Setaram C80D). The samples were outgassed at RT under ultrahigh vacuum conditions for 12 h. A detailed description of the method can be found elsewhere.^32^

### 2.4 Humidity Adsorption Tests

The water adsorption capacity of the LiCl@AC composites at each studied RH was quantified by weighing a specific amount of each sample (50–100 mg) at defined time intervals, after exposing the LiCl@AC composite to a controlled RH. The samples were placed in sealed chambers maintained at 25 °C with controlled RH, achieved using glycerol/water solutions, and continuously monitored by a calibrated digital Hygro-Thermometer RS PRO (model RS 325), Figure S1b. Target RH levels were set based on glycerol/water ratios reported in the literature.^33^ The RH sensor calibration procedure is described in detail in the Supporting Information.

Before conducting the humidity tests, pristine AC, LiCl@AC composites, and UiO-66 were thermally treated in an oven at 150 °C for 12 h, while zeolite 13× was treated at 250 °C for 12 h. Once placed inside the sealed chamber with monitored humidity, samples were left for 24 h or until adsorption equilibrium was reached. During testing, RH fluctuations were within ±2 °C. After completing the humidity tests, the LiCl@AC with greater water uptake, UiO-66 and zeolite 13× were subjected to identical regeneration studies carried out at 40 °C, 80 °C, and 100 °C for 3 h in an oven. Adsorption and regeneration tests were triplicated.

## 3 Results and Discussion

### 3.1 Characterization of LiCl@AC Composites

The textural properties of pristine AC are critical for achieving a high dispersion of the hygroscopic salt, and developing an optimal gas–solid interface necessary to maximize moisture adsorption. RGC-30 granular activated carbon (designated as AC) was selected as the platform material due to its high *S*_BET_ of over 1300 m^2^/g and its well-developed micro and mesoporous structure. N_2_ adsorption/desorption isotherms at cryogenic temperature of AC exhibit a type IIb isotherm with a narrow knee at low relative pressures, which is characteristic of microporous AC. The desorption curve differs from the adsorption curve, forming an H-type hysteresis loop at high relative pressures due to condensation in the mesoporous region (Figure 1a). The isotherm confirms the presence of a highly developed micro and mesoporous structure with a total pore volume of up to 1.12 cm^3^/g. LiCl@AC composites exhibit a similar profile, reflecting the combined presence of micro- and mesopores. However, a slight decrease in the total amount of N_2_ adsorbed is observed as the LiCl loading increases, with the effect being more pronounced at low-relative pressure (*P*/*P*_0_ < 0.2). In the pristine AC, the *S*_BET_ is 1359 m^2^/g and the total pore volume is 1.12 cm^3^/g; these values decrease to 776 m^2^/g and 0.43 cm^3^/g, respectively, when 30 wt % LiCl is incorporated (Table 1). Regarding the pore size distribution (PSD) data (Figure 1b), derived from DFT analysis of N_2_ adsorption isotherms, reveal distinct peaks at ≈0.8, 1.1, and 3.2 nm. These PSD data show a clear trend: as the LiCl loading increases, the number of those pores consistently decreases. This is strong evidence that the incorporation of LiCl preferentially blocks the micropores.

**Figure 1:**
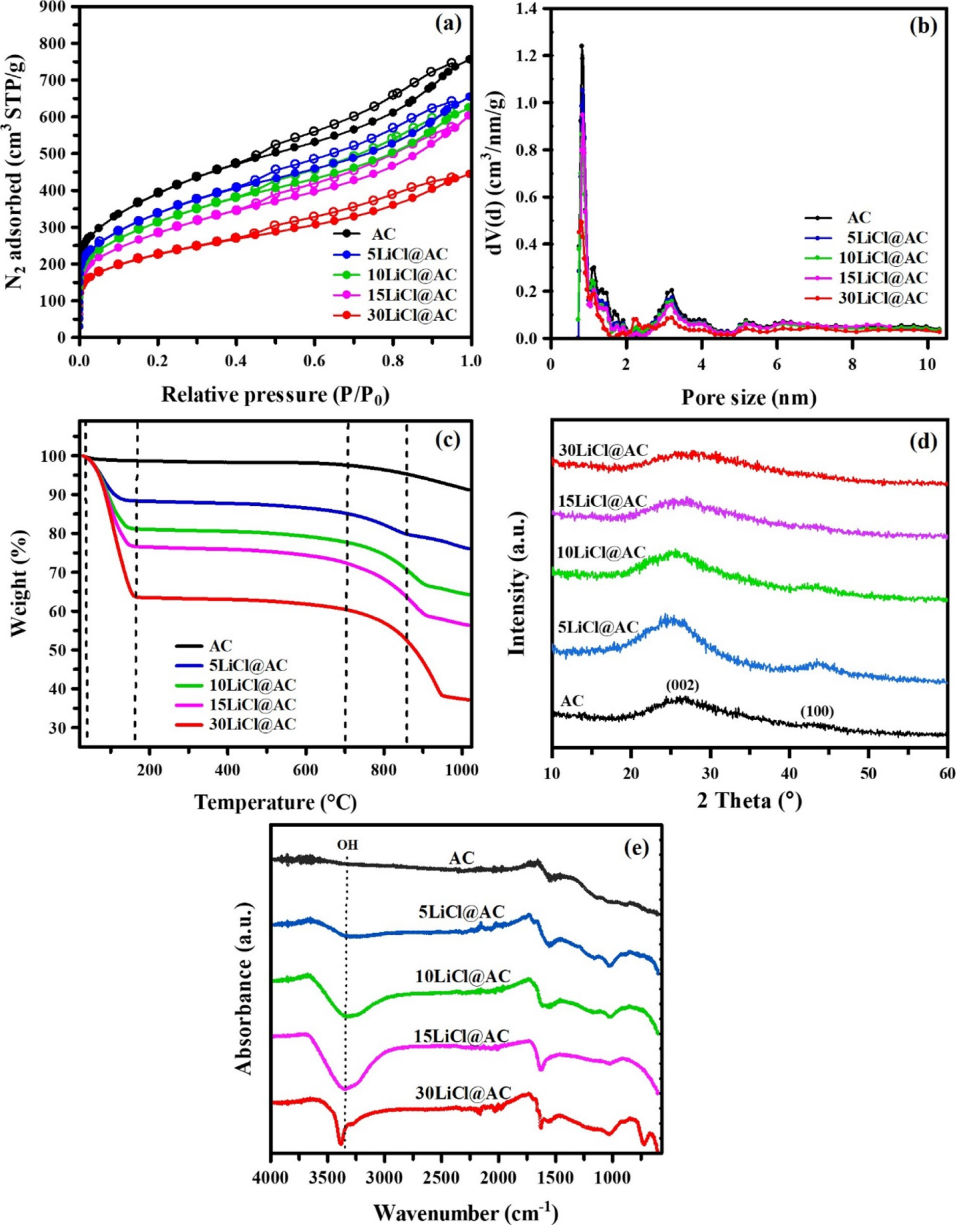
(a) N_2_ adsorption (filled symbols)/desorption (open symbols) isotherms at 77 K for the AC and LiCl@AC composites, (b) pore size distribution (PSD) determined by DFT method, (c) thermogravimetric analysis under an inert atmosphere (N_2_) in the temperature range of 25 to 1025 °C, (d) XRD diffraction patterns of LiCl@AC composites, and (e) FTIR spectra of LiCl@AC composites.

**Table 1:** Textural Parameters Determined by N_2_ Adsorption at 77 K, Li Content before Use and Post-Cycling, and Calorimetric Immersion Values of the LiCl@AC Composites

Sample	*S*_BET_ (m^2^/g)	*V*_total_ (cm^3^/g)	*V*_micro_ (cm^3^/g)	*V*_meso_ (cm^3^/g)	Li content (mg/kg)	Li content (mg/kg) postcycling	–Δ*H*_imm_ (J/g)
AC	1359	1.12	0.66	0.45	-----	------	27
5LiCl@AC	1144	0.95	0.43	0.52	13,245.0	13,101.0	107
10LiCl@AC	1103	0.92	0.40	0.52	27,819.7	27,248.9	148
15LiCl@AC	995	0.86	0.36	0.50	35,747.3	33,905.1	201
30LiCl@AC	776	0.43	0.19	0.24	54,926.6	42,548.5	324

The thermogravimetric behavior of the synthesized samples was evaluated over a temperature range of 25 to 1025 °C using N_2_ as inert atmosphere (Figure 1c). As expected, weight losses in the original AC are minimal (approximately 3–4% at the maximum temperature), with two distinct regions: 25 to 200 °C and 700 to 950 °C. The first region corresponds to the loss of moisture, while the second is likely related to the stability of the AC sample and the potential decomposition of some surface functional groups. After the incorporation of the hygroscopic salt, the thermogravimetric curve follows a similar profile, showing two main weight losses in the same temperature intervals, with the magnitude of these changes being proportional to the amount of salt incorporated. It is well-known in the literature that LiCl is a very stable salt, with a melting point above 600 °C.^34^

The first weight loss, observed at 30 °C is associated with the desorption of water molecules from the hydrated salt, reflecting the moisture adsorption capacity of the composites after the incorporation of the LiCl crystals.^35^ The amount adsorbed by the as-synthesized samples is 1 wt % (AC), 4.1 wt % (5LiCl@AC), 9.3 wt % (10LiCl@AC), 13.8 wt % (15LiCl@AC), and 27.6 wt % (30LiCl@AC). This amount corresponds to the total humidity adsorbed by the composites several weeks after preparation. The perfect linear relationship between the adsorbed humidity and the salt content indicates the success of the impregnation step. Furthermore, except for pristine AC, all profiles exhibit a second step above 700 °C, attributed to the decomposition and evaporation of the molten salt.^34,35^ Interestingly, the presence of carbon improves the stability of LiCl, shifting the decomposition temperature from 850 °C in the sample with 5 wt %, up to 950 °C for the samples with 30 wt %. The final degradation products could be either Li_2_O_2_ or Li_2_O.^34,36^ Although the salt content in the synthesized samples cannot be precisely estimated from the thermogravimetric analyses, the observed linearity suggests that the actual salt content is likely close to the nominal value.

Figure 1d presents the X-ray diffraction patterns of the LiCl@AC composites, it is observed a broad peak reaching its maximum at 2θ = ∼26° attributed to the (002) plane of disordered, nongraphitic carbon, which reflects the largely amorphous nature of the material. A much weaker, more diffuse peak associated with the (100) plane appears around 43° 2θ, this suggests the presence of a small number of graphite structures. It is also evident that increasing the LiCl loading results in a noticeable attenuation of the intensities of these two main peaks. However, no distinct reflections corresponding to either anhydrous or hydrated LiCl are visible in the diffractogram. This absence suggests that LiCl is likely well-dispersed within the pores or micropores of activated carbon, without crystallizing or forming very small crystals, hence, its characteristic peaks of LiCl may be too weak to distinguish from the amorphous carbon background.

The hydrophilicity of the LiCl@AC composites was evaluated through FTIR analysis (Figure 1e), where the O–H stretching band (≈3350 cm^–1^) became progressively broader and more intense with increasing LiCl loading, consistent with the enhanced interaction of water molecules with hygroscopic LiCl salts confined within the porous structure of the activated carbon matrix. It is expected that most of the LiCl salt is inside the porous rather than on the external surface.

The morphology of the synthesized samples was examined using FESEM imaging. Figure 2 presents representative images of pristine AC and LiCl-modified samples. The FESEM images reveal the presence of dispersed white LiCl crystals that are homogeneously distributed throughout the modified samples. The concentration of these LiCl crystals increases proportionally with the metallic salt content. Additionally, its presence was confirmed by the EDS maps, which exhibit the distribution of C, O, and Cl elements. Lithium (Li) is a light element with low X-ray emission energy, thus it is not detectable using standard EDS.

**Figure 2:**
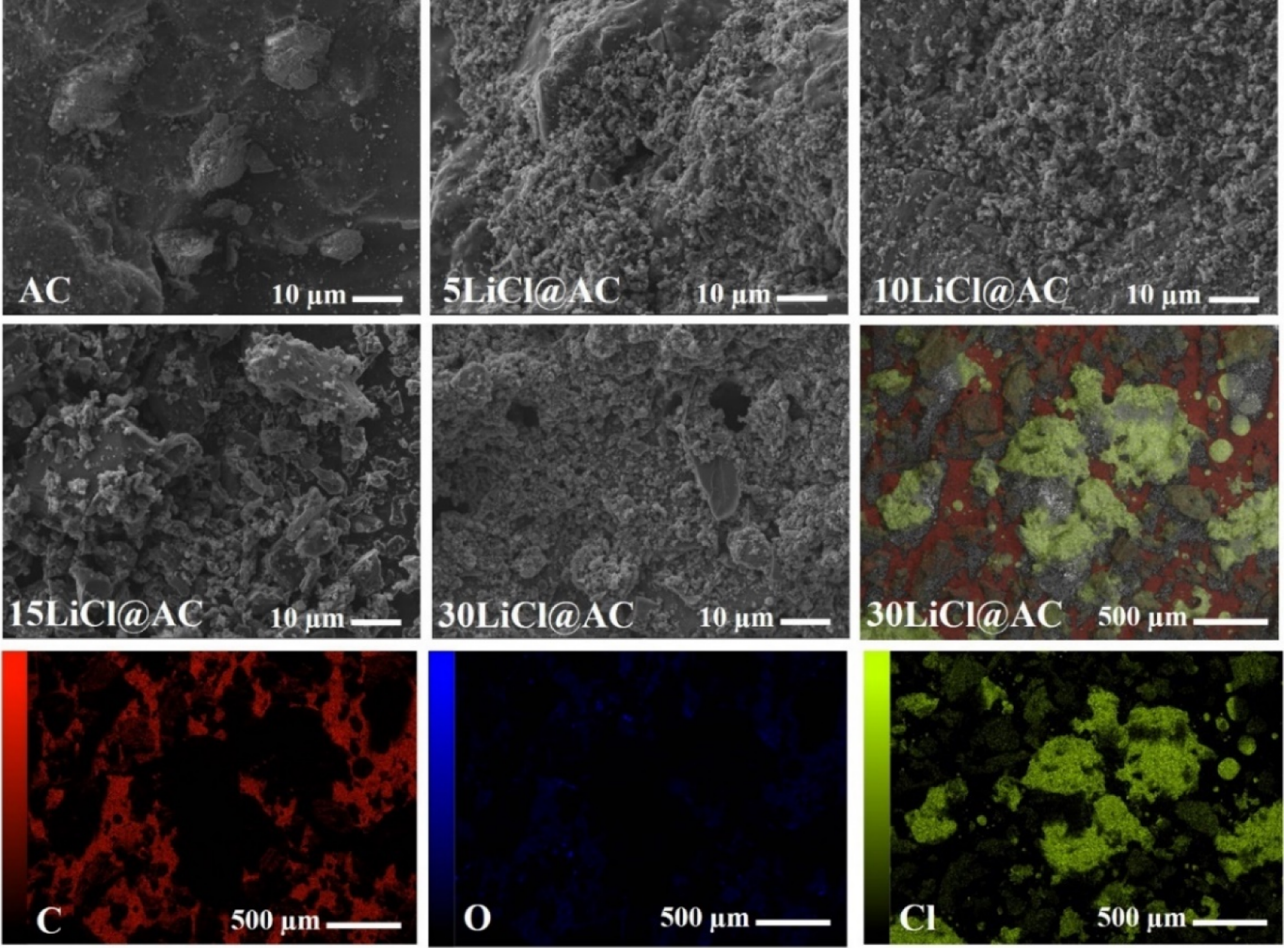
FESEM images of the AC and LiCl@AC composites and EDS elemental mapping showing the C, O, and Cl distribution on 30LiCl@AC sample.

The ICP analysis was employed to determine the presence of Li in the composites, the results are shown in Table 1. As expected, the Li content ranged from 13,245.0 mg/kg in the sample with the lowest LiCl content (5LiCl@AC) to 54,926.6 mg/kg in the sample with the highest LiCl content (30LiCl@AC), in close agreement with the nominal values, thus confirming the effective incorporation of LiCl into the AC during the impregnation process.

To further characterize the physicochemical and hygroscopic properties of the synthesized composites, immersion calorimetry measurements were conducted using water as a probe molecule. This technique quantifies the enthalpy of immersion (−Δ*H*_imm_) of each sample in a defined liquid, providing insights into the sample’s wettability, the accessibility of the probe molecule to the internal porous structure, and the presence of specific liquid–solid interactions at the interface. Table 1 reports the enthalpy values for the LiCl@AC composites treated solely under ultrahigh vacuum conditions. The main goal was to identify preferential interactions between the AC sample and water molecules after incorporating hygroscopic salts, as well as to assess the extent of these interactions. The enthalpy of immersion in water for pristine AC is as low as 27 J/g, in agreement with the hydrophobic nature of the AC sample.^37,38^ Incorporating the hygroscopic salt significantly increases the enthalpy value, reaching a maximum of 324 J/g for the 30LiCl@AC composite, 12 times higher than that of pristine AC. Although this value is notably high for a hydrophobic sample like AC, it is comparable to the enthalpies reported for zeolites when using water as a probe (e.g., 300–350 J/g, for zeolites 4A and 5A).^32^ Overall, these results confirm a substantial enhancement in water-framework interactions in hydrophobic carbon materials following LiCl impregnation, with an increase of over an order of magnitude. The perfect linear correlation between −Δ*H*_imm_ (J/g) and the LiCl content further confirms the success of the impregnation process and the optimal accessibility of water molecules to the hygroscopic active sites.

### 3.2 Water Sorption Performance

A key question at this stage is quantifying the amount of moisture the synthesized samples can retain under different environmental conditions and assessing their cyclability. To address this, the samples were tested under varying RH levels, and the adsorbed moisture was quantified based on weight changes. In all evaluated scenarios, water uptake reaches equilibrium with 1000 min. Only at high RH (60%) LiCl@AC composites require more time to reach equilibrium (over 3000 min) compared to UiO-66 and Ze13X. Water uptake kinetics for 30%, 40%, and 60% RH are reported in Figure 3.

**Figure 3:**
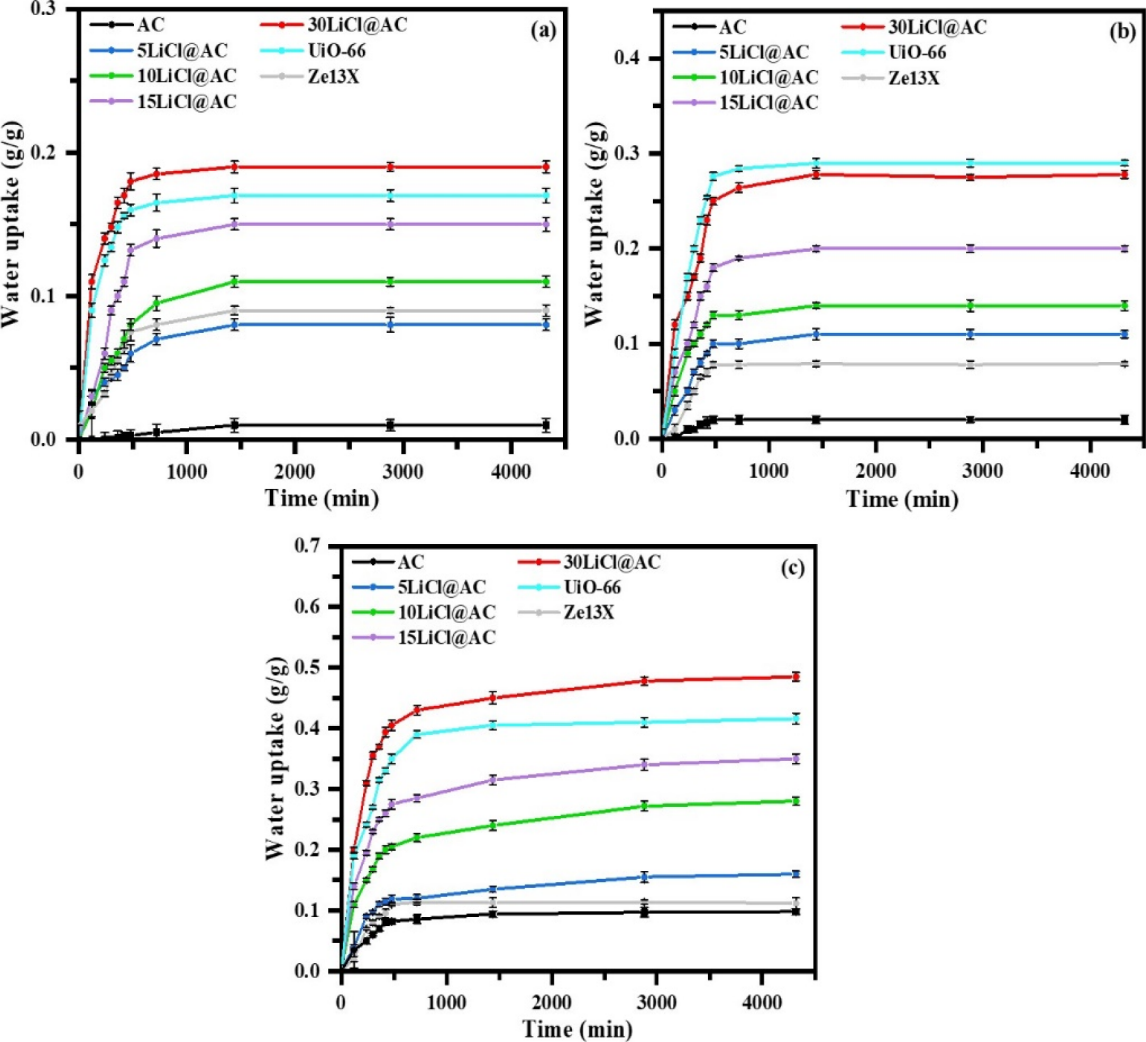
Water uptake kinetics for (a) 30%, (b) 40%, and (c) 60% RH at 25 °C.

Figure 4a shows the amount of water uptake per unit mass of LiCl@AC composites at RH levels of 30%, 40%, 60%, and 90%. As expected, water uptake increased with higher relative humidity and greater LiCl content in the samples. The most favorable conditions, in terms of moisture adsorption, were observed at 90% RH and 25 °C. Under these conditions, the total uptake ranged from 0.43 g/g, for pristine AC, to 1.3 g/g, for the sample modified with 30 wt % LiCl. Incorporating LiCl into a porous matrix within an optimal concentration range enhances the hygroscopic performance of the adsorbent. Studies have demonstrated that increasing LiCl content up to approximately 30 wt % improves water uptake capacity and adsorption kinetics.^39,40^ However, further increases in LiCl concentration can lead to salt aggregation, which may obstruct pore accessibility and reduce the effective surface area for moisture adsorption. This aggregation can diminish the overall hygroscopic efficiency of the composite material.

**Figure 4:**
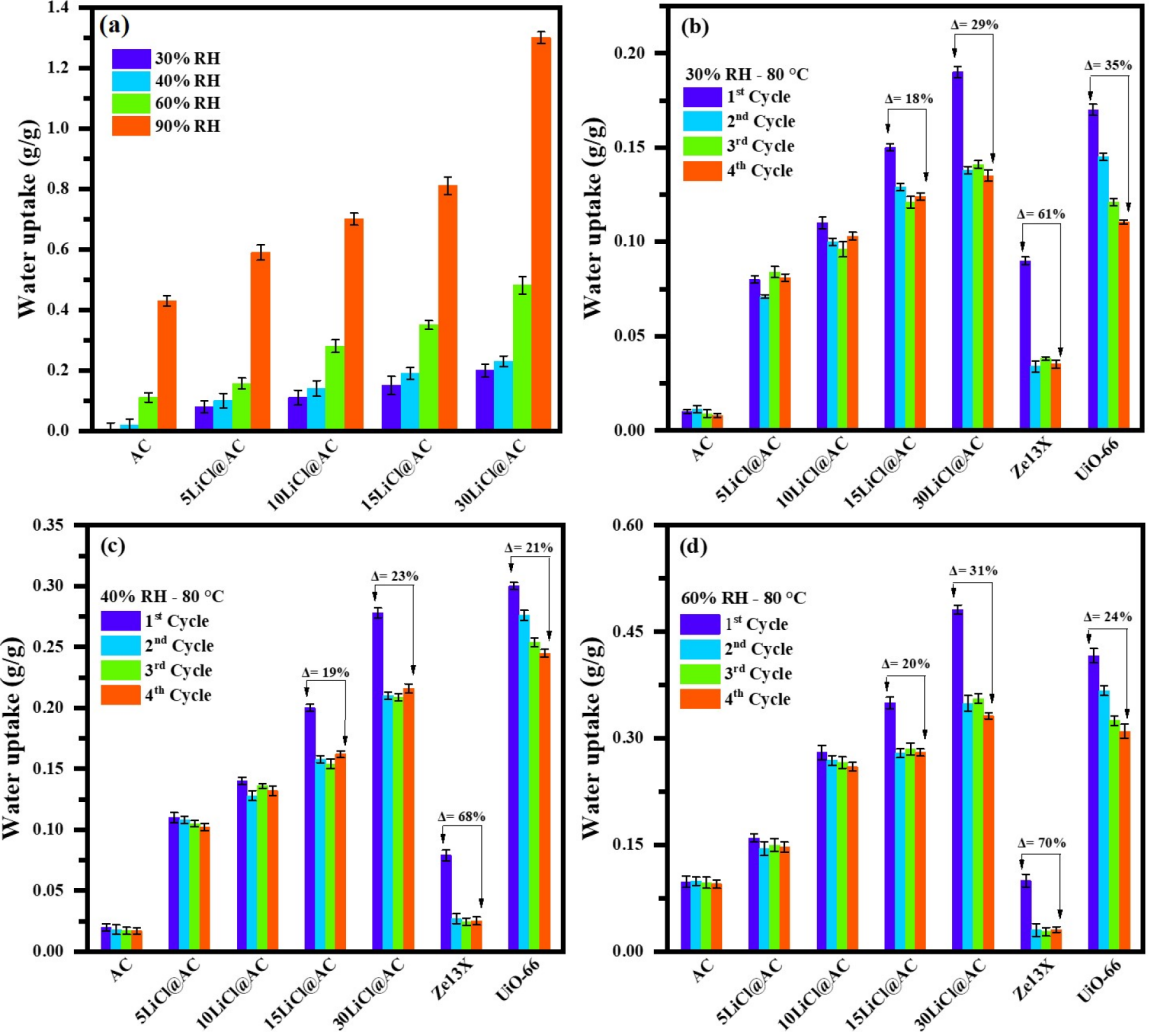
(a) Water uptake of AC, LiCl@AC composites, Ze13X, and UiO-66 under varying RH conditions (30–90%) at 25 °C. Consecutive sorption/desorption cycles of the materials at (b) 30% RH, (c) 40% RH, and (d) 60% RH, evaluated with a regeneration treatment at 80 °C for 3 h.

Pure LiCl was not evaluated under identical relative humidity (RH) conditions in this work because previous research by Zhao et al. demonstrated that pure LiCl exhibits significant water adsorption across various RH levels due to its strong water affinity. Nevertheless, LiCl tends to deliquesce even at low RH values, such as 35% RH.^21–23,29,39^ This behavior underscores the importance of incorporating LiCl into a porous matrix.

Even so, the current challenge lies in developing sorbents capable of functioning effectively under harsh conditions, particularly at humidity levels similar to those found in end-user locations, such as the Mediterranean region, where RH is typically below 60%. Under these conditions, the performance of the pristine AC is very limited, with water uptake around 0.11 g/g at 60% RH, 0.02 g/g at 40% RH, and nearly zero adsorption at 30% RH. These results suggest that AC materials with poor surface chemistry are ineffective for trapping humidity at low RH, despite their highly developed porous structure. Fortunately, this scenario changes drastically with the incorporation of LiCl. At 60% RH, the adsorbed amount increases to 0.16 g/g, 0.28 g/g, 0.35 g/g, and 0.48 g/g for the modified samples with 5, 10, 15, and 30 wt % LiCl, respectively. The water uptake for the sample containing 30% LiCl is 4 times higher than that of pristine AC. Even under harsh conditions (30% RH), the 30LiCl@AC sample can adsorb 0.19 g/g. A review of the literature reveals that the performance of these LiCl-based composites is highly promising. The uptake values up to 0.48 g/g at 25 °C and 60% RH are above those reported for some zeolites and MOFs.^8,19,29^

The usefulness of a sorbent for application in water harvesting devices depends on its ability to maintain performance after multiple cycles. To evaluate the cyclability of the best-performing material (30LiCl@AC), Ze13X, and UiO-66, the samples were first thermally treated in an oven at 80 °C for 3 h after each adsorption cycle and then retested under different environmental conditions. Figure 4b–d compares the water adsorption capacity after 4 consecutive cycles for the evaluated samples at 3 selected humidity levels: 30%, 40%, and 60% RH. For comparison, zeolite 13X (designated as Ze13X) and the metal–organic framework UiO-66 were included in the analysis. As previously observed in Figure 4a, water uptake increases with both salt content and RH. Notably, at low humidity levels (30% RH), the sample 30LiCl@AC demonstrates significantly better performance compared to Ze13X and UiO-66. The total amount adsorbed under these conditions reaches 0.19 g/g, Figure 4b.

Regarding the regeneration step at 80 °C, both pristine and modified AC achieve complete regeneration after four consecutive cycles for salt loadings below 10 wt %. However, for higher salt loadings, regeneration is incomplete, with this effect becoming more evident as the salt content increases. Incomplete regeneration refers to a progressive decrease in adsorption capacity (within an experimental deviation of ±5%), suggesting partial water retention or limited desorption efficiency at a given temperature, in this case at 80 °C. Moreover, postcycling ICP-OES analysis (Table 1) revealed a decrease in Li content for all LiCl@AC composites, confirming partial salt loss after the fourth regeneration cycle. The Li loss was more pronounced at higher loading (>10 wt %). Composites with 5%, 10%, and 15% LiCl lost approximately 1%, 2%, and 5% of their Li content, respectively, whereas the sample containing 30 wt % LiCl exhibited a significant loss of 22%. Moreover, microscopy images (SEM) reveal morphological changes (Figure S2). As the LiCl loading increases, the salt no longer appears as well-defined crystals deposited on the surface or within visible pores. Instead, the morphology consistently shifts toward a more homogeneous appearance—almost “melted-in” or integrated into the carbon matrix—showing much less evidence of sharp crystalline edges. This suggests that the salt is partially dissolved, or that crystals have been so finely dispersed or altered by moisture exposure that they are no longer distinguishable as macroscopic crystalline entities.

For the 30LiCl@AC sample at 30% of RH, the decrease in water uptake from the first to fourth cycle is approximately 29%. While this reduction is notable, it is significantly smaller than the losses observed for Ze13X (61%) and UiO-66 (35%). At 40% and 60% RH, the LiCl@AC samples and UiO-66, exhibit an average capacity loss of around 19–31% after 4 cycles. Specifically, the 15LiCl@AC composite shows a lower capacity loss than UiO-66, while the 30LiCl@AC composite experiences a slightly higher loss. In contrast, regeneration for Ze13X remains a challenge under the same conditions. A closer look of the regeneration step for highly loaded samples (e.g., 15LiCl@AC and 30LiCl@AC) reveals that the capacity loss is significant after the first regeneration cycle, with subsequent cycles having minimal additional impact. The same behavior is observed for Ze13X. This can be attributed to one of the following reasons: (i) the loss of salt after the first cycle, although this does not apply to Ze13X and UiO-66; (ii) blockage by residual water molecules that are not fully removed at 80 °C, an effect more pronounced in the hydrophilic zeolite sample; or (iii) decreased H_2_O–H_2_O interactions, which are necessary to promote clustering of water molecules. A thorough review of the literature indicates that LiCl evaporation takes place at temperatures exceeding 800–1000 °C.^35,41^ However, it is also well-known that LiCl has a high solubility in water (up to 84.25 g/100 mL at 25 °C) owing to the hygroscopic nature of the small Li^+^ cation. While the loss of some salt during the thermal treatment at 80 °C cannot be completely ruled out, any such loss would likely be minimal and insufficient to account for the observed regeneration losses. Furthermore, the consistent regeneration performance observed from the second cycle onward rules out the possibility of LiCl crystal agglomeration (high cycling stability), which is typically observed in bulk LiCl crystals.^8^ Based on these observations, the most plausible explanation for the reduced water uptake after the first regeneration cycle is the presence of residual water molecules adsorbed within the LiCl crystals, indicating incomplete regeneration. Modified H_2_O–H_2_O interactions after the regeneration step at 80 °C for 3 h can be excluded, as the samples were pretreated at 150 °C for 3 h before the first cycle.

Overall, the results confirm that under relatively dry atmospheric conditions (RH 30%), AC materials modified with LiCl were greater than Ze13X and UiO-66 in terms of water uptake and regeneration capability. Higher humidity levels further enhance the adsorption performance and regeneration capacity, particularly for LiCl@AC and MOF samples. However, the regeneration step at 80 °C appears insufficient to fully regenerate the sorbent, an effect that is more pronounced at low humidity levels and during the first cycle, especially for samples with high salt loadings. This observation is consistent with the presence of intracrystalline water molecules (monohydrated LiCl), which are not easily desorbed at 80 °C. After the second cycle, the amount of occluded H_2_O seems to remain constant.^42^ Moreover, the regeneration results in Figure 4b–d suggest that the impact of intracrystalline occluded water is only significant for high LiCl loadings, likely due to the formation of larger crystals. The poor regeneration observed for Ze13X at 80 °C aligns with the strong hydrophilic nature of zeolites and their high affinity for water.

To assess the effect of the regeneration step, similar experiments were conducted with the best-performing material, 30LiCl@AC, and for comparison purposes, UiO-66 and Ze13X. These regeneration tests were performed at a lower regeneration temperature (40 °C for 3 h) with samples pre-exposed to 30% and 60% RH conditions. Figure 5a,b shows the water uptake of four consecutive cycles.

**Figure 5:**
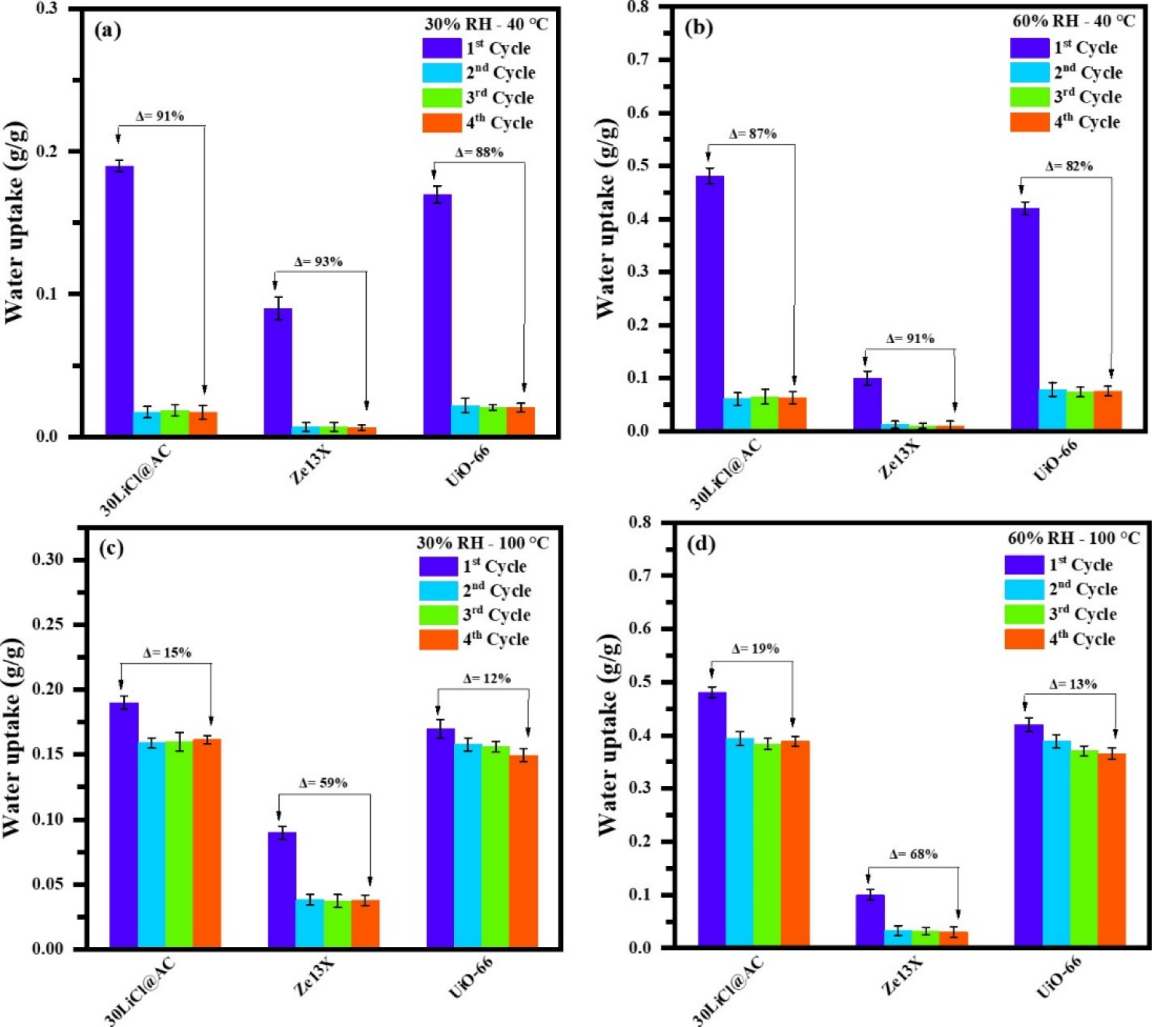
Water adsorption capacity at 30% RH and 60% RH, for the best-performing material (30LiCl@AC), Ze13X, and UiO-66 after regeneration treatment at (a,b) 40 °C, and (c,d) 100 °C.

The regeneration results clearly demonstrate that 40 °C is a very low temperature for all samples, regardless of their nature (carbon-based composites, zeolites, or MOFs). Water uptake losses range from approximately 82–93% when comparing the first and fourth cycles, with the most significant decrease occurring after the first cycle, as previously noted. Additional cycling tests were conducted at a regeneration temperature of 100 °C for 3 h (Figure 5c,d). The 30LiCl@AC and UiO-66 samples showed superior regeneration capabilities, with water uptake losses ranging between 12% and 19%. The slight decline in performance for the 30LiCl@AC composite after the first cycle could be attributed to residual water molecules that are not completely desorbed, UiO-66 exhibited slightly better cyclability performance. However, Ze13X, still requires a higher regeneration temperature to achieve effective desorption. These findings confirm that 80 °C can be considered the minimum temperature required for effective regeneration of the carbon-based composites.

Additionally, water uptake after the first cycle was compared for different carbon-based composites and normalized by the total salt content. Figure 6 shows that the water content per gram of LiCl is approximately 0.63–2.2 g/g_salt_ for all carbon-based samples evaluated at 30% and 40% RH. However, this amount scales up to ca. 3.2 g/g_salt_ at high humidity (60%) conditions. This result clearly proves that hygroscopic salts work more efficiently at high humidity. The decreased efficiency with salt content 5 wt % > 10 wt % > 15 wt % > 30 wt % must be related to the decreased salt dispersion at high loadings. In a molar basis, water uptake per salt content (mol/mol_salt_) ranges from 1.5 to 5.2 at 30% and 40% RH, respectively, while this value scales up to 7.5 mol/mol_salt_ for sample 5LiCl@AC at 60% RH (Figure S3). Taking into account that LiCl is usually present as mono-, tri- and pentahydrate salt, these results suggest the preferential presence of monohydrated LiCl crystals at low humidity values, while at high humidity (60%), the high efficiency can only be explained by the participation of the carbon support in the adsorption process.

**Figure 6:**
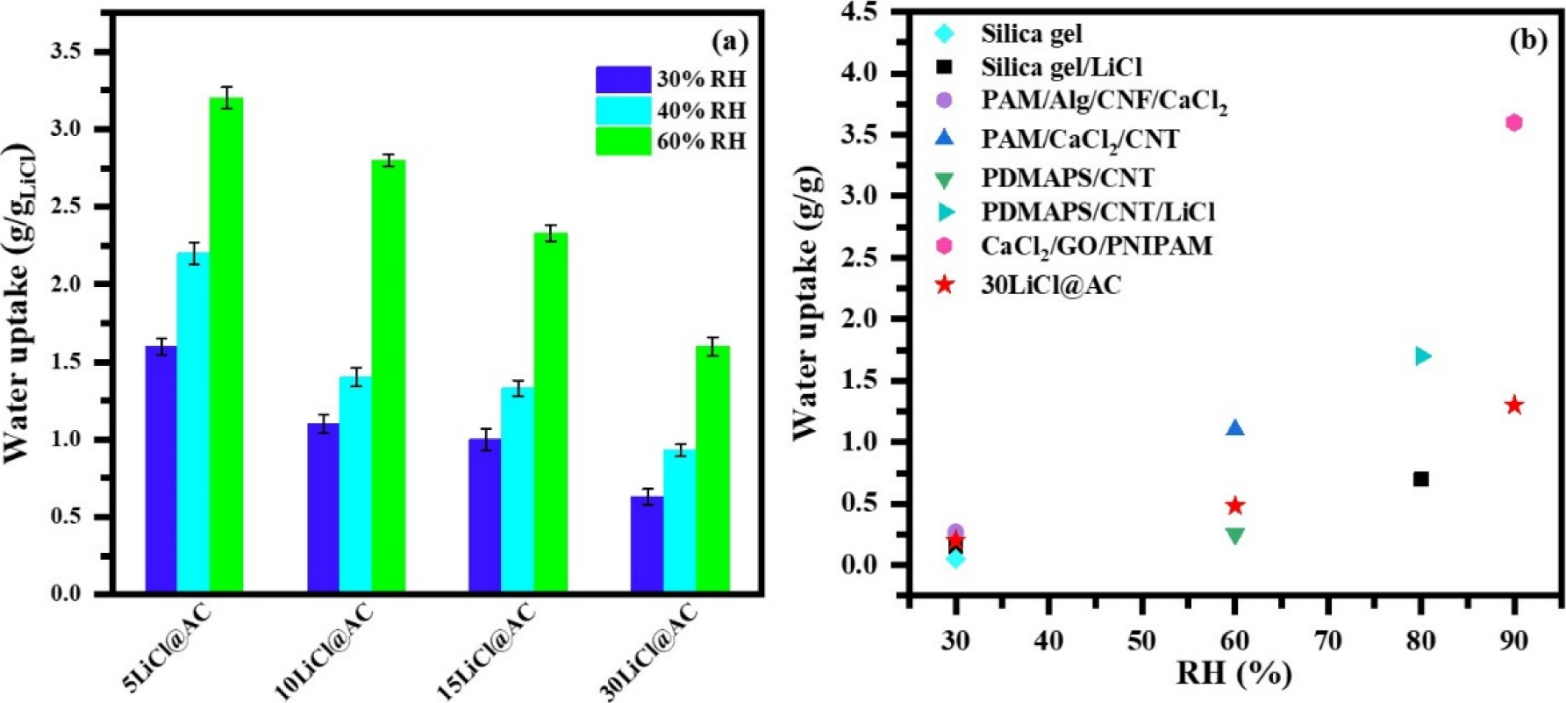
(a)­Water uptake (g/g_salt_) normalized by the nominal salt content for all carbon-based composites evaluated at 30% RH, 40% RH, and 60% RH, and (b) Benchmark against commercial desiccants and emerging materials.

Figure 6b compares the water uptake of LiCl@AC composites with that of commercial desiccants (e.g., silica gel) and with hydrogels as emerging materials. Overall, hydrogels exhibit superior performance across all tested RH conditions (Table 2), whereas the silica gel shows lower water uptake than 30LiCl@AC. For instance, CaCl_2/_GO/PNIPAM hydrogel achieved 3.2 g/g at 90% RH, while the 30LiCl@ AC composite reached 1.3 g/g under the same RH. Under low-RH conditions (e.g., 30% RH) a silica gel/LiCl attained 0.15 g/g, compared to 0.19 g/g for 30LiCl@ AC. Although the LiCl@AC composites do not exhibit the highest water uptake when compared to advanced materials such as hydrogels, they offer several practical advantages. The preparation method is simple, does not require specialized synthesis steps, and uses low-cost and commercially available raw materials. Because of these characteristics the composites are highly attractive for large-scale applications, where ease of manufacturing, cost-effectiveness, and operational simplicity are often more important than maximum water adsorption capacity. Therefore, these composites represent a viable and scalable option for real-world humidity control systems, especially under economic and energy constraints.

**Table 2:** Water Adsorption Capacity of Commercial Desiccants and Emerging Materials

Material	RH (%)	Water uptake (g/g)	Reference
Silica gel	30	0.05	^13^
Silica gel/LiCl	30	0.15	^13^
30LiCl@AC	30	0.19	this work
PAM/AlG-CNF/CaCl_2_	30	0.27	^43^
PAM/CaCl_2_/CNT	60	1.10	^44^
PDMAPS/CNT	60	0.25	^45^
30LiCl@AC	60	0.48	this work
Silica gel/LiCl	80	0.70	^13^
PDMAPS/CNT/LiCl	80	1.70	^45^
CaCl_2/_GO/PNIPAM	90	3.60	^46^
30LiCl@AC	90	1.30	this work

Figure 7 depicts the schematic of the adsorption–desorption process in the LiCl@AC composite. When water vapor encounters the composite, the molecules interact with LiCl particles within the pores, initiating hydration and forming crystalline LiCl·H_2_O in the solid state. As hydration progresses, deliquescence occurs: the solid hydrates develop a thin surface film of saturated salt solution. Simultaneously, remaining solid LiCl continues to absorb water until it is fully converted into a saturated LiCl solution. During desorption, the composite is heated, causing release of water vapor and restoring the material for the next cycle.

**Figure 7:**
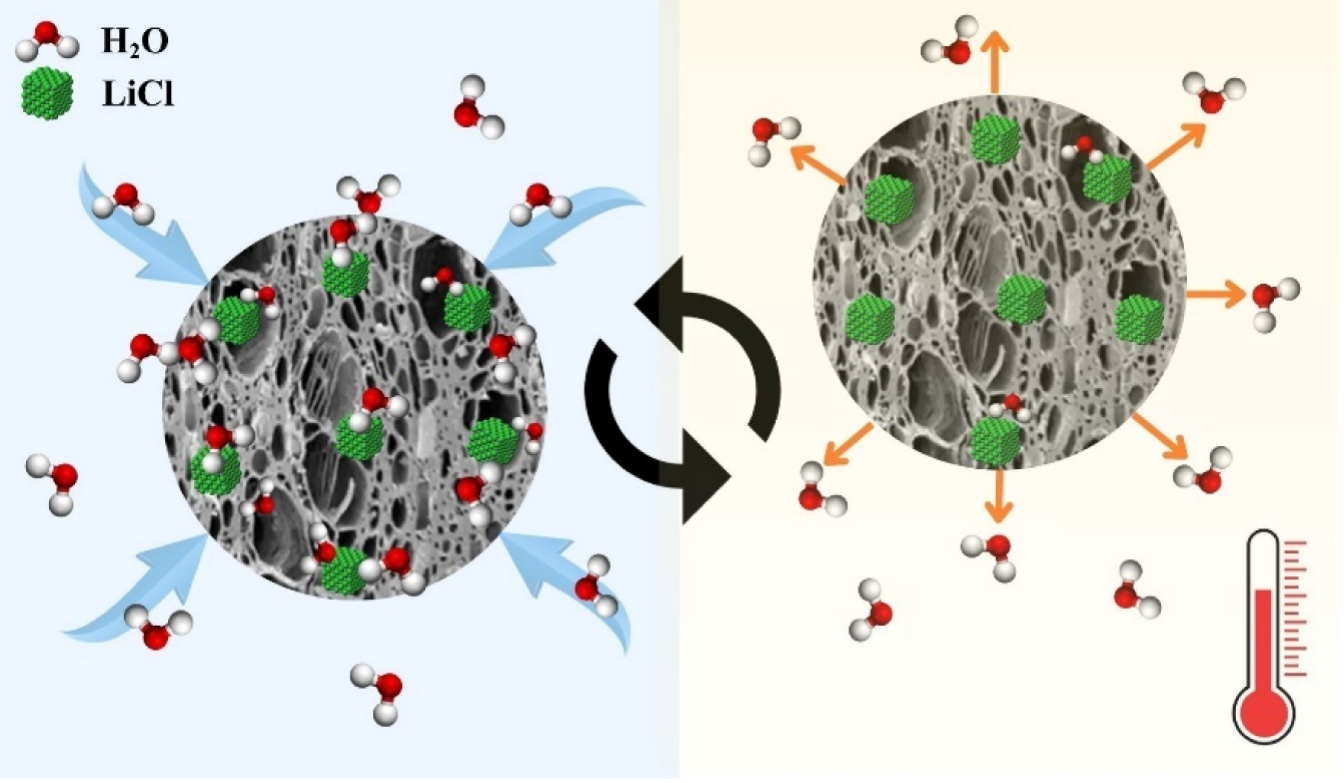
Schematic illustration of the adsorption–desorption process in the LiCl@AC composite.

In addition to the water uptake and cycling stability demonstrated by the LiCl@AC composites, practical considerations such as material safety and energy requirements must be addressed for real-world deployment.

In 2021, the U.S. Environmental Protection Agency categorized the Li as the fifth unregulated pollutant. Li meets the criteria for hormonal disrupters, affecting the thyroid, hepatic, and nervous functions and potential environmental impact warrant careful consideration. Although the lethal concentration 50% (LC_50_) has been studied for some aquatic organism, it has been suggested that these toxicological effects may be potentialized by other factors.^47^

In terms of the energy requirements, water desorption is a determining factor for practical implementation. LiCl-based sorbents regenerated at 80–90 °C, reported energy requirements range from 0.25 to 0.65 kWh/L of produced water, which is significantly lower than that of seawater reverse osmosis (2.5 to 4.0 kWh/m^3^) for modern plants.^48^ Some studies mention that with efficient energy recovery, real-world plants may approach lower values, though pre- and post-treatment and system inefficiencies push the total toward or above ∼3 kWh/m^3^.^49^

These findings suggest that sorption-based AWH systems could provide an energy-efficient and sustainable alternative for decentralized freshwater production, particularly in arid and coastal Mediterranean regions.

## 4 Conclusions

A series of carbon-based composites were prepared by incorporating the hygroscopic salt LiCl into the pores of activated carbon with a well-developed porous structure. The confinement of LiCl crystals within the activated carbon pores was intended to reduce the common drawbacks of inorganic salts, such as deliquescence, agglomeration, and solution leakage, while enhancing the hydrophilicity of the composites. The results confirm that under relatively dry atmospheric conditions (30% RH), activated carbon materials modified with hygroscopic salts are superior in terms of water uptake and cyclability than Ze13X and UiO-66. Among the composites, 30LiCl@AC exhibited the higher water uptakes of 0.11 g/g, 0.25 g/g, 0.48 g/g, and 1.3 g/g at 25 °C under 30%, 40%, 60%, and 90% RH, respectively. Cyclability results suggested that the impact of intracrystalline occluded water became significant at higher LiCl loadings, likely due to the formation of larger crystals. Furthermore, due to the strong interactions between water molecules and the LiCl@AC composites, 40 °C as regeneration temperature was insufficient (the minimal temperature required was 80 °C).

## Supplementary Material



## Notes

Figure S1 (a-b) Methodology for the preparation and performance of LiCL@AC; sensor calibration procedure; Figure S2 Postcycling SEM images; Figure S3 Water uptake (mol/mol_salt_) (PDF)
