# Supplementary material for: LiCl@AC Composites for Atmospheric Water Harvesting: Effect of the Salt Content
Source: ACS Omega. 2025 Nov 5;10(45):54329–40. doi: 10.1021/acsomega.5c06858 (PMC12631357; doi:10.1021/acsomega.5c06858)
Supplement: Supplementary file 1 [file ao5c06858_si_001.pdf]

LiCl@AC composites for atmospheric water harvesting: Effect of the salt content

Sonia Judith Segovia-Sandoval[1*], Antonio García-Ripoll,[1] Judit Farrando-Perez,[1] Coset Abreu-Jauregui,[1] Joaquin Silvestre-Albero[1*]

[1] Laboratorio de Materiales Avanzados, Departamento de Química Inorgánica-Instituto Universitario de Materiales, Universidad de Alicante, Spain

[*]Corresponding authors: Sonia Judith Segovia-Sandoval [1*], Joaquín Silvestre-Albero[1*]

SUPPORTING INFORMATION

Figures	Content	Page
**Fig. S1**	• Scheme for the (a) preparation of the LiCl@AC composites. Impregnation of activated carbon with LiCl solution and subsequent filtration and drying to obtain the final composites (b) Humidity tests.<br>• RH sensor calibration procedure	S2
**Fig. S2**	SEM images from LiCl@AC composites post-cycling	S3
**Fig. S3**	Water uptake (mol/mol_{salt}) normalized by the nominal salt content for all carbon-based composites evaluated at 30% RH, 40% RH, and 60% RH.	S4

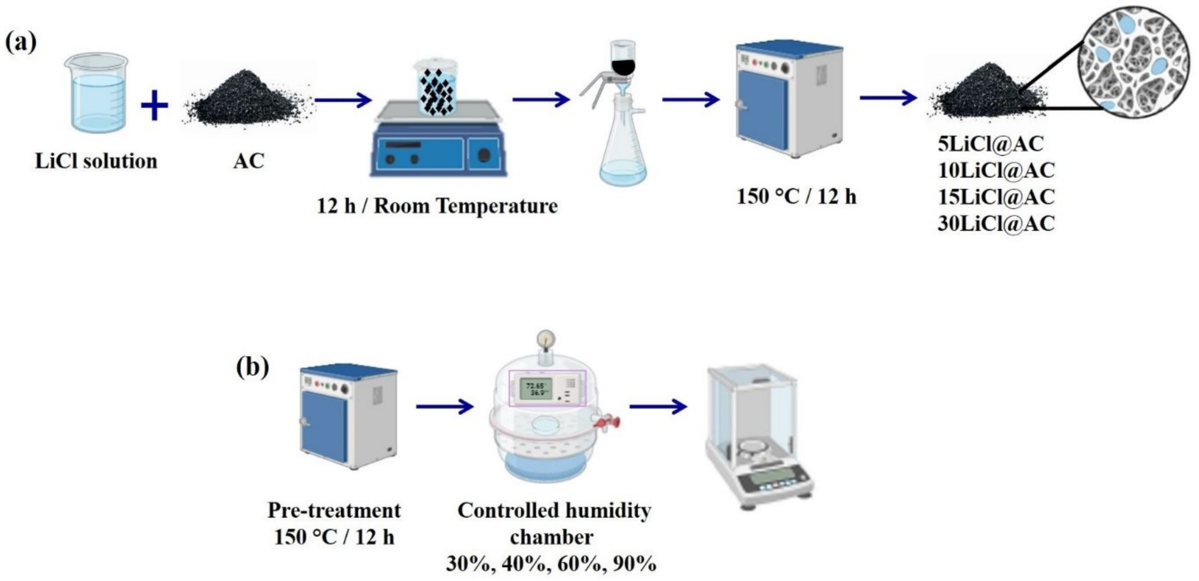

Fig. S1. Scheme for the (a) preparation of the LiCl@AC composites. Impregnation of activated carbon with LiCl solution and subsequent filtration and drying to obtain the final composites (b) Humidity test.

RH sensor calibration procedure:

Prior to use, the reliability of the RH sensors was validated using saturated salt solutions as reference points. The employed salt solutions used to cover a wide range of humidity were $MgCl_2$ ≈33 % RH, NaBr ≈58 % RH, NaCl ≈75 %, KCl ≈87 % RH at 25 °C, prepared and equilibrated in sealed chambers according to ASTM E104 [1]. After the equilibrium was reached (24-48 h), sensor readings were recorded and compared to the reference values [2]. The results showed a maximum deviation of ± 2% RH across the tested range.

References

[1] Standard Practice for Maintaining Constant Relative Humidity by Means of Aqueous Solutions (E104-20a). ASTM International.

[2] R.Weast, CRCHandbook of Chemistry and Physics 1976-1977:A Ready-Reference Book of Chemical and Physical Data, 57th ed. (CRC, Boca Raton, 1976).

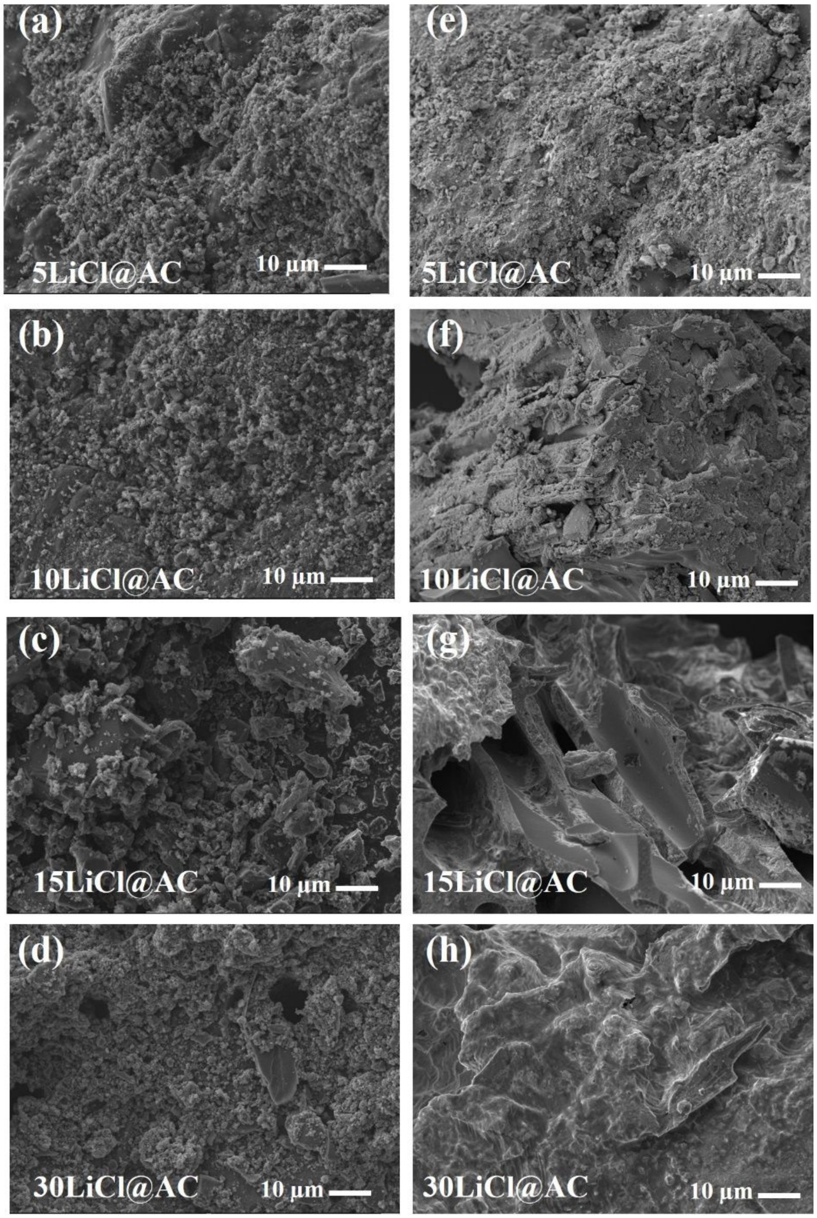

Fig. S2. SEM images (a-d) LiCl@AC composites before use, and (e-h) after the fourth regeneration cycle.

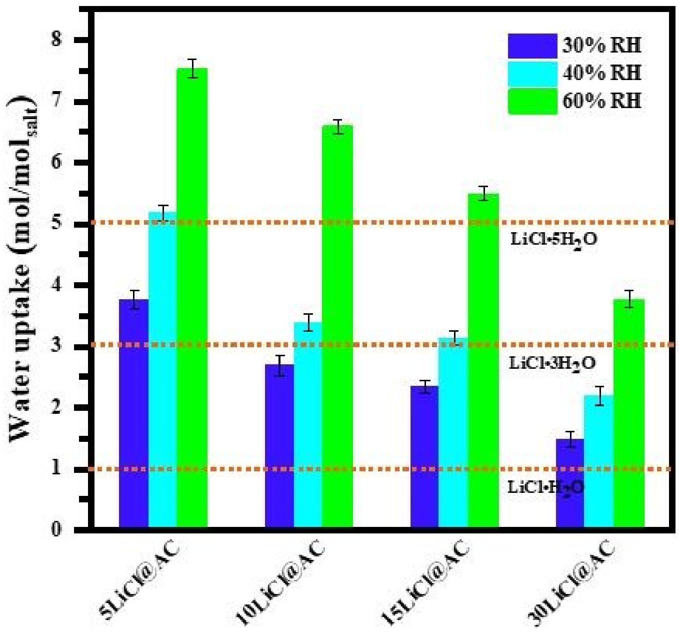

Fig. S3. Water uptake (mol/mol$_{salt}$) normalized by the nominal salt content for all carbon-based composites evaluated at 30% RH, 40% RH, and 60% RH.
